# A direct comparison of laboratory and community EEG recordings for neurodevelopmental research

**DOI:** 10.1038/s41598-025-03569-5

**Published:** 2025-07-02

**Authors:** Abigail Dickinson, Summer-Rose Perry, Amanda Gulsrud, Connie Kasari

**Affiliations:** University of California, Los Angeles (UCLA), Los Angeles, CA USA

**Keywords:** Neuroscience, Psychology, Translational research

## Abstract

Leveraging portable electroencephalography (EEG) to measure brain function in community settings offers a promising strategy to improve the scalability and accessibility of developmental neuroscience research. To encourage broader adoption of these methods, it is important to demonstrate that data quality and neural signal integrity are comparable to gold-standard lab-based recordings. In this study, we directly compared EEG data collected in laboratory and home environments using portable EEG systems in a developmentally diverse group of young children under four years of age (n = 10). Despite differences in equipment and setting, our results showed comparable data quality and signal characteristics across conditions. Specifically, EEG data retention rates, noise levels, and spectral power measures were highly consistent at the group level, with no systematic differences between lab- and home-based recordings. To assess individual-level consistency, we calculated intraclass correlation coefficients (ICCs) for spectral power across brain regions and frequency bands. Most region-by-band combinations showed good to excellent consistency across settings; however, lower consistency was observed for some lower-frequency metrics, such as delta power in parietal regions. This suggests that certain individual features may be more sensitive to contextual or developmental factors. Overall, our findings demonstrate that portable, community-based EEG maintains data quality and neural signal integrity comparable to laboratory systems. Broader use of portable EEG may enhance scalability, increase participation, and promote greater inclusion in neurodevelopmental research.

## Introduction

Electroencephalography (EEG) is a powerful, non-invasive tool for measuring brain function in developmental populations. By capturing voltage changes from neuronal firing with millisecond precision, EEG provides real-time insights into synchronized neural oscillations that underlie functional brain circuits^1,2^. Combined with its tolerability in infants and young children, this high temporal resolution has made EEG an essential method for mapping oscillatory rhythms during early brain development^3,4^. In clinical settings, EEG is routinely used to monitor brain activity in neonatal intensive care units, where it helps to detect seizures, assess brain injury^5,6^, and predict long-term developmental outcomes in preterm infants. Moreover, EEG has been instrumental in identifying neural signatures of altered brain function in diverse contexts, including neurodevelopmental disorders^7–9^, genetic conditions^10–12^, and the effects of early adversity such as prenatal substance exposure^13^ and institutional care^14^.

However, leveraging EEG to track early brain development at a broader population level requires more scalable and accessible collection methods. EEG is typically collected using high-density systems in controlled clinical or research environments. These methods yield high-quality data, but can limit accessibility for families facing transportation challenges, inflexible work schedules, or caregiving responsibilities, which disproportionately affect under-resourced and racially/ethnically diverse communities^15–19^, limiting the generalizability and impact of findings.

Recent advancements in portable EEG technology provide promising solutions by enabling data collection in community-based settings, including homes and schools. Innovations such as active electrodes, which enhance signal-to-noise ratio, and noise-shielding technology allow these systems to maintain data quality outside laboratory environments^20,21^. Leveraging portable systems for community-based EEG collection could make research participation more accessible and enable measures of brain function in naturalistic settings where children are more comfortable, potentially capturing neural activity that better reflects real-world cognitive and behavioral processes.

To support broader use of these methods, it is critical to establish that community-based EEG yields data comparable to traditional lab-based recordings, especially in young children who present unique challenges such as shorter attention spans and increased movement artifacts^22,23^. In adult populations, several studies have demonstrated comparable signal quality between portable EEG collected in non-lab settings and traditional recordings, with no significant differences in spectral power or noise^24–27^. For example, one study found no significant differences in alpha and beta power between lab-based and outdoor EEG recordings^24^, while another reported comparable alpha power and minimal electromyographic (EMG) interference when comparing home and lab settings^27^. While prior research has demonstrated the feasibility of recording EEG from children in homes and schools^28–31^, direct comparisons with lab-based EEG recordings remain unexamined in developmental populations.

Our study addresses this gap by directly comparing EEG recordings obtained in both community-based and lab-based settings among young children under four years of age. This sample represents a broad range of developmental trajectories without excluding participants based on neurodevelopmental status, ensuring our findings are applicable to diverse populations. To evaluate whether community-based EEG can serve as a valid alternative to lab-based recordings in developmental research, we assess potential differences in key signal characteristics, including power in specific frequency bands, signal quality, and noise levels, across both settings. By systematically comparing these metrics, we aim to determine if portable EEG can reliably match the standards of lab-based EEG recordings, ultimately broadening the reach and impact of EEG research in developmental science through more scalable and ecologically valid methods.

This study addresses that gap by directly comparing EEG recordings collected in both community and lab settings in a developmentally diverse group of children under four years of age. By including children with a range of neurodevelopmental profiles, we aim to ensure that our findings reflect the variability encountered in real-world research contexts. To evaluate the viability of community-based EEG, we systematically compare key data collection and signal quality metrics—including frequency-specific power, noise levels, and data retention—across both settings. Our goal is to determine whether portable EEG systems can produce neural data comparable to lab-based systems, ultimately advancing the scalability, inclusivity, and ecological validity of EEG research in developmental neuroscience.

## Methods

### Participants

Eleven participants, aged six months to four years, were recruited from an existing database as part of a pilot project funded to explore promising short-term research directions (1R56DC021174-01). The UCLA Institutional Review Board (IRB) approved all study procedures, and a parent or guardian provided written, informed consent in accordance with the Declaration of Helsinki. All methods, hypotheses, and analytic plans were pre-registered prior to data analysis (bit.ly/4hiE8vN).

EEG sessions were scheduled within 30 days of each other whenever possible, allowing flexibility for family schedules. Two families opted to complete their community EEG visit before the in-lab session. All 11 participants completed the community EEG; however, one family later declined the in-lab visit due to hesitations about attending a formal research institute. Thus, our analysis includes data from the ten participants who completed both community and lab EEG sessions (Median Age: 2.73; Range: 1.65–3.36 years).

As shown in Table 1, the sample represented a diverse range of families. Less than half (40%) reported household incomes above $80,000, and 60% received government assistance through programs such as the Special Supplemental Nutrition Program for Women, Infants, and Children (WIC), the Supplemental Nutrition Assistance Program (SNAP), and Medicaid. Participants represented varied developmental trajectories, with half having an autism diagnosis and the other half undergoing evaluation for developmental concerns at the time of participation.

**Table 1:** Demographic and sample characteristics (n = 10).

Demographic variable	*n*	Percentage or Median (SD)
Age		2.73 (0.56)
Sex		
Female	3	30%
Male	7	70%
Race/ethnicity		
Asian	1	10%
Black	3	30%
White	2	20%
Hispanic	4	40%
Neurodevelopmental concerns		
Undergoing evaluation	5	50%
Autism diagnosis	5	50%
Household income		
< $50,000	5	50%
$60,000-$80,000	1	10%
> $80,000	4	40%
Maternal education (≥ some college)	5	50%
Receiving assistance (e.g., WIC, SNAP)	6	60%

### EEG collection

Continuous EEG data were acquired under two protocols: (1) using a high-density system in the laboratory and (2) using a portable EEG system in community settings. As outlined above, we aimed to obtain community and lab EEG recordings within 30 days of each other, with 90% of the final sample (n = 9) completing the lab EEG before the home visit. On average, recordings were conducted 18.2 days apart (median = 11 days, SD = 27.03).

We used standardized procedures across both recordings, aiming to obtain five minutes of continuous EEG under task-free conditions, consistent with standard practices for spontaneous (task-free) EEG recordings in developmental populations^32,33^. If a child became fussy, we extended the session to ensure adequate data collection. In-lab EEG was collected using our standard protocol, and portable EEG procedures were established to be as consistent as possible. In all cases, EEG was recorded while the participant sat on a parent’s or caregiver’s lap. For all recordings, EEG data were sampled at 1000 Hz, with electrode impedances maintained below 100 kΩ, aligning with widely used thresholds for infant populations^34^ and consistent with our established EEG protocols for developmental populations^35^. Setup times were not directly compared due to differences in electrode systems (129 channels in the lab vs. 32 in the community). However, active setup time (electrode placement and impedance checks) were consistently under 10 min for all recordings.

In-lab EEGs were collected in a soundproofed, electrically shielded room with participants seated on a caregiver’s lap. Data were collected using a 129-channel HydroCel Geodesic Sensor Net (Electrical Geodesics Inc., Eugene, OR) along with a Net Amps 300 amplifier, and Net Station 4.4.5 software. An appropriately sized HydroCel Sensor Net was soaked in saline and placed on the participant’s head, where we adjusted electrode positions and added saline as needed. Data were referenced to the vertex (Cz) during recording, and four electrooculogram (EOG) sensors positioned beneath and next to the eyes were removed from the electrode setup to improve comfort.

Community EEG recordings were conducted in locations selected by families, using published recommendations for non-laboratory EEG to guide our protocol^30,32^. Eight families opted to complete the recording at their own home or the home of a close friend or relative, while two chose community-based settings, including a facility offering activities for children and a structured day program. EEG data were recorded from 32 active gel-based electrodes (BrainProducts actiCAP slim active gel electrodes) using a BrainVision LiveAmp amplifier and BrainVision Recorder software. After measuring the participant’s head circumference, electrodes were inserted into an appropriately sized BrainProducts ActiCap with 10–20 positions (Fp1, Fz, F3, F7, FT9, FC5, FC1, C3, T7, TP9, CP5, CP1, Pz, P3, P7, O1, Oz, O2, P4, P8, TP10, CP6, CP2, Cz, C4, T8, FT10, FC6, FC2, F4, F8, Fp2). Two additional channels served as the online reference (FCz) and ground (AFz). Conductive gel was applied to the electrodes before placing the cap on the participant’s head, with extra gel added using a blunt syringe as necessary.

### EEG processing

Offline data processing was conducted using EEGLAB^33^ and custom MATLAB scripts (The MathWorks, Inc., Natick, MA). Given that lab-based EEGs were collected with fixed 129-channel caps, when importing data we selected 32 channels that aligned with the portable system’s electrode positions, consistent with previous comparison protocols^25^. All subsequent data processing steps were conducted using identical procedures and parameters for both lab-based and portable system recordings. First, we applied a finite impulse response (FIR) high-pass filter to remove frequencies below 1 Hz, followed by an assessment of 60 Hz main line noise by examining spectral power and calculating a signal-to-noise ratio (SNR). Power estimates were computed using EEGLAB’s ‘spectopo’ function with standard parameters. To derive the SNR metric, we first normalized the power spectrum for each channel to between 0 and 1 using min–max scaling. We then calculated the SNR as the ratio of the normalized power at 60 Hz to the average normalized power across the 1–50 Hz frequency range. After calculating noise metrics, a low-pass FIR filter was applied to remove frequencies above 50 Hz using EEGLAB’s default setting, which designs the filter using a Hamming window and an automatically determined filter order.

Consistent with our previous studies, we used artifact subspace reconstruction (ASR)^36^ as the primary cleaning method for all subsequent EEG analyses^37–39^. ASR was implemented using EEGLAB’s clean_rawdata function with default parameters and a channel rejection threshold of 0.7, consistent with our prior work^36–39^. However, to assess whether our estimates of data quality (i.e., the amount of data retained following artifact removal) were robust to the choice of cleaning algorithm, we also calculated data quality metrics using two additional cleaning methods: amplitude-based thresholding and manual artifact rejection.

The amplitude-based thresholding approach was adapted from our prior evaluations of multi-site infant EEG quality^39^. We used the erplab toolbox function *pop_continuousartdet*^33,40^, to remove data sections where more than 25% of channels exceeded ± 600 µV, as well as channels where over 25% of data points surpassed this threshold. We then eliminated channels deviating ± 250 µV for over 25% of an infant’s resting recording and any segments where over 25% of channels deviated ± 250 µV. This approach effectively removed artifacts surpassing established thresholds, aligning with previous evaluations of infant EEG data quality^32^. The third method involved manual rejection, where data were visually inspected to eliminate segments affected by artifacts. Although this approach is less automated and more subjective, it allows for more nuanced noise detection^30^. Data retention was evaluated as an indicator of data quality by calculating the percentage of retained data following artifact removal. Metrics for each cleaning algorithm included the ratio of clean seconds (retained data length /original data length) and channels (retained channels/32).

### Power spectral density calculation

Clean EEG data were divided into continuous three-second segments to avoid discontinuities. For each participant, we randomly selected 30 three-second segments, ensuring that an equal data amount contributed to the power calculations^41^, with 90 s meeting established thresholds shown to obtain reliable power estimates^42^. Power spectral density (PSD) was computed using MATLAB’s ‘pwelch’ function, with a window duration of 2 s and a 50% overlap. PSD analysis focused on four regions of interest: frontal, central, parietal, and occipital. Each region represented the average of three electrodes: frontal (F3, Fz, F4), central (C3, Cz, C4), parietal (P3, Pz, P4), and occipital (O1, Oz, O2), aligning with regions examined in our previous developmental EEG studies^43^. Absolute power estimates were converted to relative power by dividing each frequency bin by the sum of the entire power spectrum. Power estimates were summed across five standard frequency bands: delta (1–4 Hz), theta (4–6 Hz), alpha (6–12 Hz), beta (12–30 Hz), and gamma (30–50 Hz).

### Statistical analysis

All analyses were performed in MATLAB and R. We used two-tailed Wilcoxon Signed-Rank tests to assess differences between lab and community conditions, including recording duration, noise estimates (60 Hz power and SNR ratio), the number of retained channels, and the duration of retained seconds. To provide a more nuanced evaluation, we computed Bayesian statistics using the brms package in R. Because a direct Bayesian Wilcoxon test is unavailable, we modeled the paired differences between conditions using a robust Student’s t-distribution, which provides a nonparametric Bayesian alternative equivalent to the Wilcoxon test. Bayes factors (BF) were derived such that BF_10_ (evidence in favor of the alternative) equals 1/BF_01_, with BF_10_ values < 3 considered weak evidence and values > 10 considered strong evidence for the alternative hypothesis^44^.

For analyses of spectral power metrics (the power within each frequency band for each region), we first adjusted for age effects due to significant developmental changes and variable intervals between community and lab recordings. We regressed out the effect of age at the time of each recording using linear regression, applied separately for each frequency band and region combination. This allowed us to isolate variance in EEG power independent of age-related effects. Statistical tests, including Wilcoxon signed-rank tests and Bayesian analyses, were then applied to the resulting residuals to evaluate differences between conditions, using identical procedures to those described above. Finally, to examine individual-level consistency in power measures across conditions, we computed intraclass correlation coefficients (ICCs) using the psych package in R. Specifically, we applied two-way mixed-effects models and report single-measure consistency and absolute agreement metrics, in line with recommended best practices^45^. ICC values were interpreted in line with established guidelines describing poor (ICC < 0.50), moderate (ICC = 0.50–0.75), good (ICC = 0.75–0.9), and excellent (ICC > 0.90) reliability^46^.

## Results

**Fig. 1:**
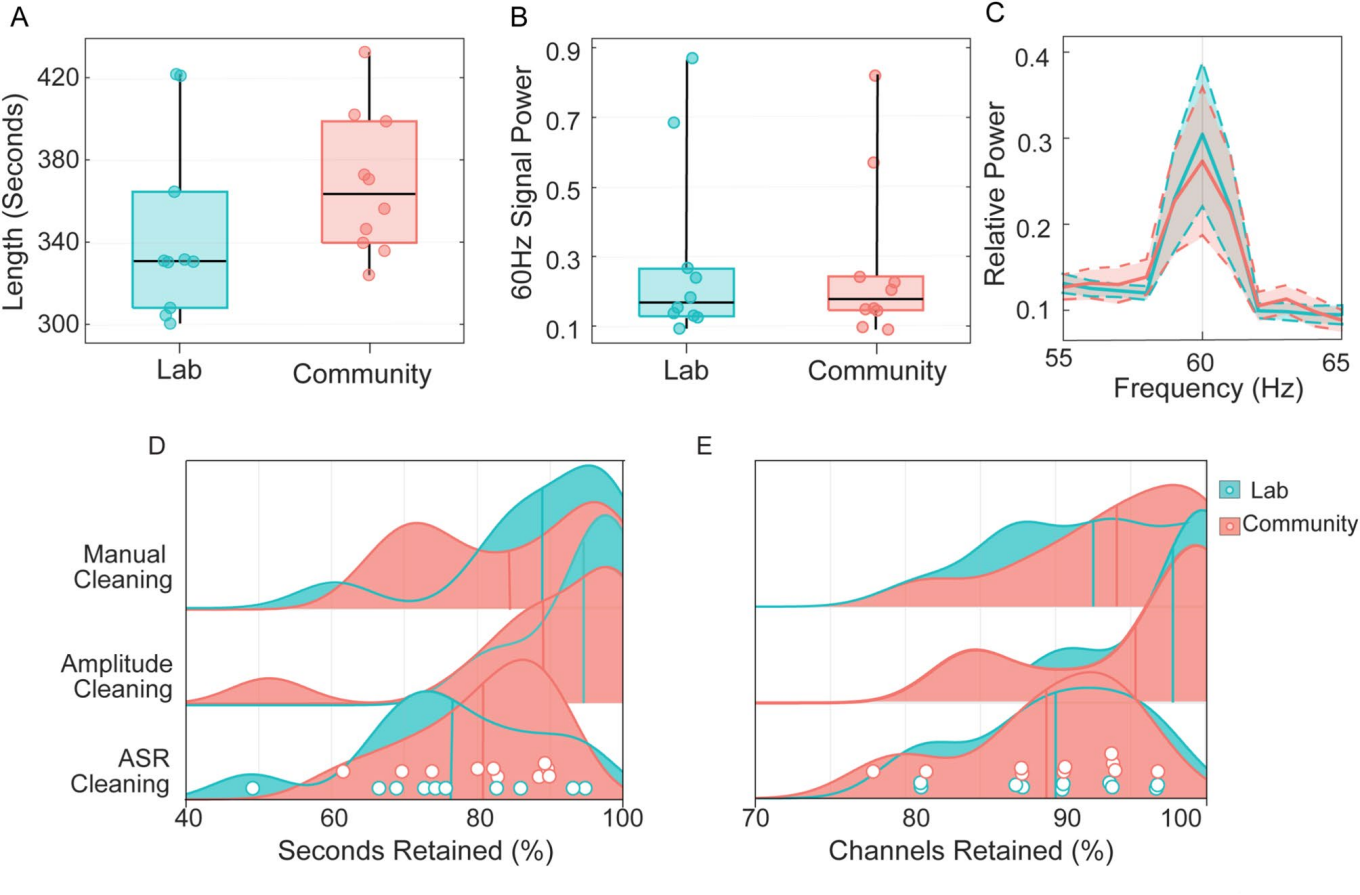
Boxplots display (**A**) recording lengths and (**B**) 60 Hz power estimates for each condition, with individual data points overlaid. (**C**) Power spectral density plotted illustrate the 60 Hz signal in each condition. Power spectra represent the average data from all 32 channels, prior to low-pass filtering and artifact removal. Shaded areas represent 95% confidence intervals. (**D**,**E**) Ridge plots depict the proportion of (**D**) seconds and (**E**) channels retained for each of the three cleaning algorithms used to assess data quality. Individual data points are overlaid to depict individual values for the primary cleaning method (ASR).

Ten participants successfully contributed EEG data in both lab and community settings, with an average recording length of 356 s. Metrics describing recording length, 60 Hz signal power, 60 Hz SNR, and data retention rates for each condition are summarized in Table 2 and Fig. 1 (A-C). Wilcoxon rank sum tests indicated no significant differences between community and lab-based recordings in the amount of data collected (W = 41, p = 0.19) or noise estimates, including 60 Hz signal power (W = 28, p = 1.00) and 60 Hz SNR (W = 24, p = 0.77). Bayesian analysis provided anecdotal evidence supporting the null hypothesis regarding 60 Hz noise estimates (BF_10_< 1). However, for recording duration, Bayesian evidence (BF_10_ = 1.41) provided anecdotal support for the alternative hypothesis, with community recordings (median: 363.46 s) being slightly longer than lab recordings (median: 330.74 s).

**Table 2:** Comparison of data collection and quality metrics: Wilcoxon signed-rank test and Bayesian analysis.

	Lab		Community		W	P	BF10
	Median, Mean (SD)	Range	Median, Mean (SD)	Range			
Recording length	330.74, 344.34 (44.5)	300.45–421.67	363.46, 367.89 (34.43)	324.02–432.46	41	0.19	1.41
60 Hz Power	0.29, 0.17 (0.27)	0.09–0.87	0.27, 0.18 (0.24)	0.09–0.82	28	1	0.82
60 Hz SNR	1.3, 0.73 (1.1)	0.54–3.37	1.27, 0.77 (1.34)	0.39–4.50	24	0.77	0.83
Cleaning algorithm 1 (ASR)							
Channels (%)	90, 90.63 (5.67)	81.25—96.88	89.38, 90.63 (5.93)	78.12–96.88	26	0.91	0.82
Seconds (%)	76.37, 75.14 (13.67)	48.90—94.82	80.76, 82.59 (9.62)	61.73–89.96	39	0.28	3.32
Cleaning algorithm 2 (amplitude thresholds)							
Channels (%)	97.81, 100 (3.91)	90.62–100.00	95.31, 98.44 (6.46)	84.38–100.00	8	0.33	0.24
Seconds (%)	94.59, 97.94 (6.81)	81.35–99.39	89.02, 93.01 (14.87)	97.94–100.00	21	0.56	0.52
Cleaning algorithm 3 (manual cleaning)							
Channels (%)	92.5, 93.75 (6.45)	81.25–100.00	94.06, 95.31 (6.15)	93.75–100.00	15	0.44	7.07
Seconds (%)	88.92, 91.85 (11.62)	60.48–98.46	84.51, 86.11 (12.55)	66.46–99.04	21	0.56	0.59

Reported p-values are derived from Wilcoxon Signed-Rank tests.

To assess data quality, we evaluated data retention rates following artifact removal (Table 2; Fig. 1D, E). Wilcoxon rank sum tests showed no significant differences in the percentage of channels or seconds retained across conditions for various cleaning algorithms, including ASR (channels: W = 26, p = 0.91; seconds: W = 39, p = 0.28), amplitude-based (channels: W = 8, p = 0.33; seconds: W = 21, p = 0.56), and manual cleaning (channels: W = 15, p = 0.44; seconds: W = 21, p = 0.56). Bayesian analysis largely supported these null findings (BF_10_ < 1) but indicated substantial evidence in favor of the alternate for two metrics: channels retained after manual cleaning (BF_10_= 7.07) and seconds retained after ASR (BF_10_ = 3.32).

**Fig. 2:**
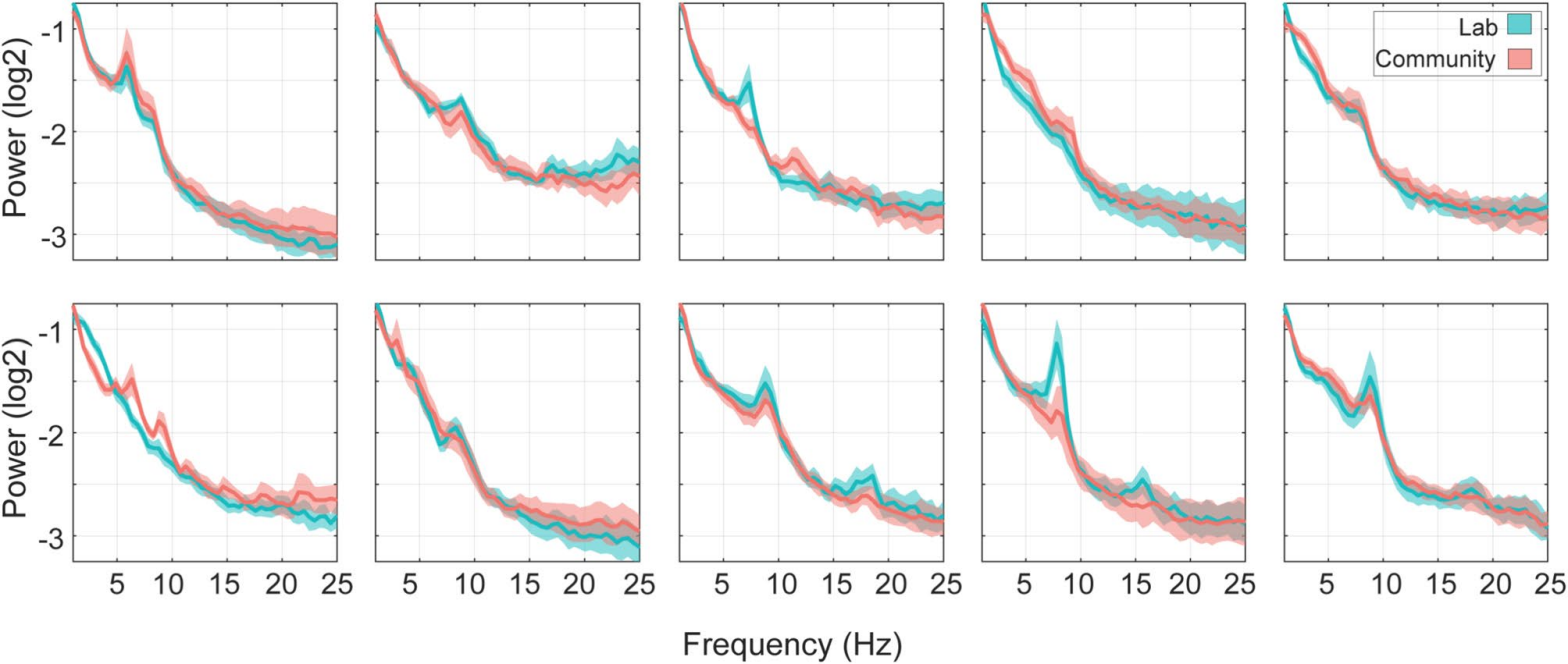
PSD plots provide participant-level comparisons of spectral characteristics. Each subplot shows the power spectra for a single participant, with lab recordings in blue and community recordings in red. The power spectra are averaged across all 32 channels, with shaded regions representing confidence intervals calculated from the standard deviation of channel values.

**Fig. 3:**
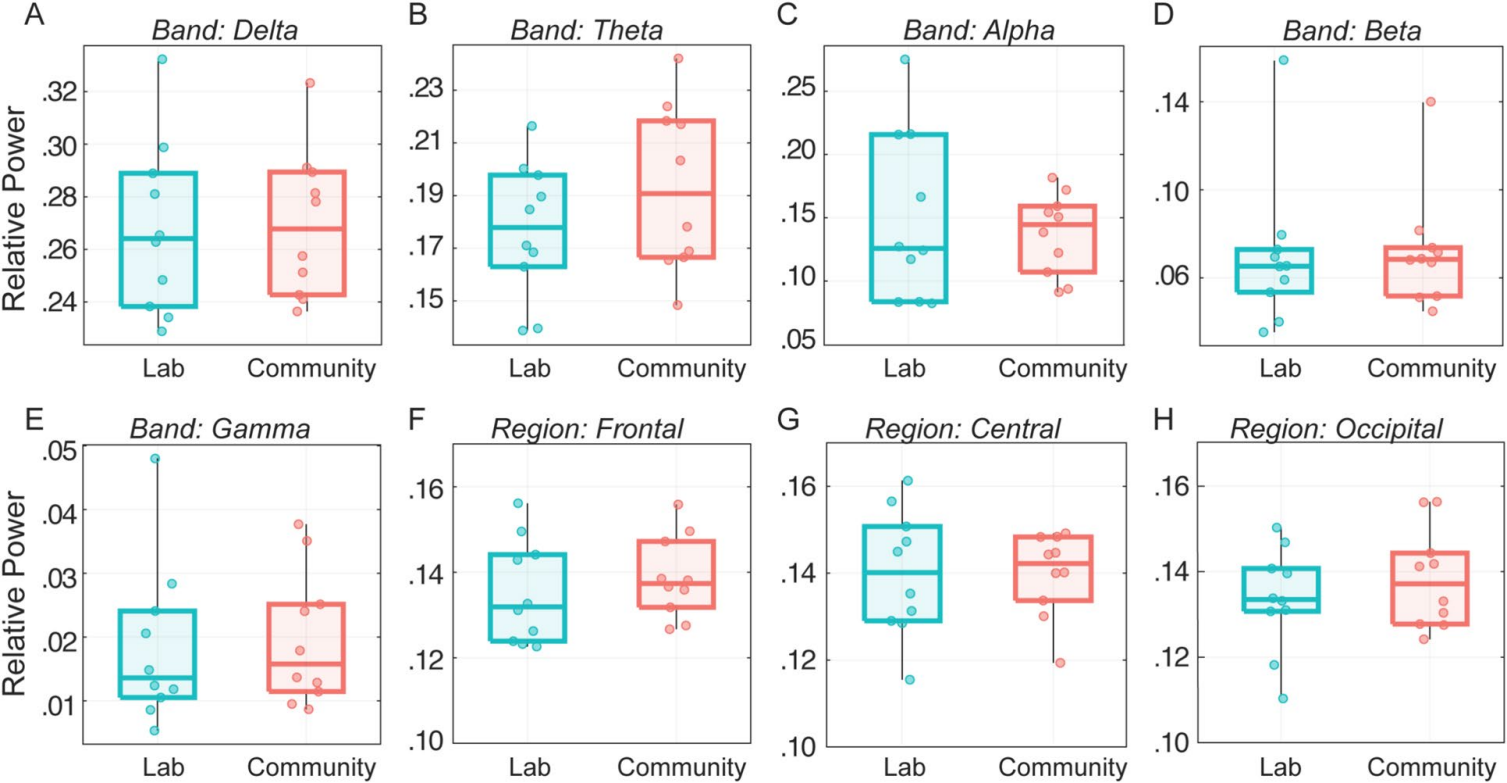
Boxplots illustrating power values across conditions. Panels (**A**–**E**) show power values for each spectral band (averaged across all brain regions), with individual participant data points overlaid. Panels (**F**–**H**) display power values for three specific regions (Frontal, Central, and Occipital), with power values averaged across all spectral bands, and individual participant data points overlaid for clarity.

Signal characteristics were examined by comparing spectral power measures. Figure 2 presents averaged PSD estimates for each participant across all 32 channels, showing that spectral characteristics were highly consistent across recording conditions. Boxplots summarizing power measures are provided in Fig. 3, with statistical analysis detailed in Table 3. Wilcoxon signed-rank tests indicated no significant differences between lab and community recordings across all spectral measures (all p > 0.44). Bayesian analyses provided further support for the null hypothesis, with the highest BF_10_ value (1.84), suggesting minimal evidence in favor of differences between conditions.

**Table 3:** Comparison of spectral power measures: Wilcoxon signed-rank test, Bayesian evidence, and Intra-class correlations (ICC).

	Lab	Community	Comparing conditions			ICC	
	Median (SD)	Median (SD)	W	P	BF10	ICC_C_	ICC_A_
Frontal							
Delta	0.26 (0.03)	0.26 (0.03)	24	0.77	1.28	0.82	0.69
Theta	0.09 (0.01)	0.09 (0.02)	26	0.92	1.2	0.16	0.09
Alpha	0.03 (0.01)	0.03 (0.01)	27	1	1.17	0.62	0.45
Beta	0.01 (0.003)	0.01 (0.002)	31	0.77	0.96	0.96	0.92
Gamma	0.002 (0.001)	0.002 (0.001)	26	0.92	1.04	0.87	0.77
Central							
Delta	0.25 (0.05)	0.26 (0.04)	26	0.92	1.28	0.13	0.07
Theta	0.09 (0.01)	0.09 (0.02)	26	0.92	1.15	0.77	0.63
Alpha	0.03 (0.02)	0.04 (0.01)	26	0.92	1.17	0.67	0.50
Beta	0.01 (0.002)	0.01 (0.001)	20	0.49	1.84	0.88	0.79
Gamma	0.002 (0.001)	0.002 (0.001)	20	0.49	1.4	0.79	0.66
Parietal							
Delta	0.27 (0.04)	0.26 (0.03)	23	0.7	1.27	0.00	0.00
Theta	0.09 (0.02)	0.1 (0.03)	26	0.92	0.96	0.87	0.77
Alpha	0.04 (0.02)	0.04 (0.01)	24	0.77	1.36	0.13	0.07
Beta	0.01 (0.002)	0.01 (0.001)	27	1	1.02	0.98	0.97
Gamma	0.002 (0.001)	0.002 (0.001)	22	0.63	1.24	0.97	0.94
Occipital							
Delta	0.29 (0.03)	0.27 (0.05)	27	1	0.96	0.82	0.69
Theta	0.09 (0.01)	0.1 (0.01)	33	0.63	0.69	0.16	0.09
Alpha	0.03 (0.01)	0.03 (0.01)	30	0.85	0.97	0.62	0.45
Beta	0.005 (0.002)	0.01 (0.001)	30	0.85	0.77	0.96	0.92
Gamma	0.002 (0.001)	0.002 (0.001)	28	1	0.9	0.87	0.77

All ICCs were computed using two-way mixed-effects models.

*W* Wilcoxon signed-rank test statistic, *ICC* intra-class correlation, *ICC*_*A*_ ICC absolute agreement, *ICC*_*C*_ ICC consistency.

On average, spectral power measures showed good consistency across conditions (median ICC_C_ = 0.81) and moderate absolute agreement (median ICC_A_ = 0.67). However, there was substantial variability across specific region-by-band combinations (see Table 3). For instance, ICC Consistency metrics, calculated using a two-way mixed-effects model, showed good/excellent reliability for beta (median = 0.96, range: 0.88–0.98) and gamma (median = 0.87, range: 0.79–0.97) bands across regions. However, average reliability was moderate for delta (median = 0.47, range: 0–0.82), mixed for alpha (median = 0.62, range: 0.13–0.67), and lowest for theta (median = 0.77, range: 0.16–0.87), with strong regional variability observed.

Agreement metrics (ICC_A_) were generally lower than consistency metrics (ICC_C_) and also showed variability across regions and frequency bands, particularly in the lower-frequency ranges. For example, beta and gamma bands showed good to excellent agreement across regions (beta: 0.79–0.97; gamma: 0.66–0.94). In contrast, alpha, theta, and delta bands showed greater variability. While alpha and theta bands demonstrated moderate agreement in several regions, some region-by-band combinations yielded poor agreement (alpha: 0.07–0.50; theta: 0.09–0.77; delta: 0.00–0.69).

## Discussion

This study directly compared lab-based and community EEG recordings in a developmentally diverse cohort, representative of the range of neurodevelopmental variability seen in real-world clinical and research settings. To assess whether community EEG is a viable alternative to traditional lab-based recordings, we systematically evaluated practical data collection metrics, including the total amount of data collected, noise estimates, and data quality. Data quality was indexed by the proportion of data removed when applying three different cleaning algorithms. We also compared signal characteristics by analyzing spectral power across different scalp regions and frequency bands.

Our data suggest that it is feasible to collect community EEG recordings of comparable quality to lab-based EEG. Specifically, we did not observe significant differences between settings in the amount of data collected, 60 Hz noise estimates, or data quality metrics. For most of these comparisons, Bayesian analyses supported the null hypothesis (BF_10_< 1), consistent with the absence of meaningful differences between lab and community EEGs. There was, however, weak evidence for a difference in recording length (BF_10_ = 1.41) and moderate evidence for differences in two data retention metrics (BF_10_ = 3.32–7.07). Importantly, upon inspecting the data, these differences consistently favored community EEG, with slightly longer recording durations and higher data retention compared to lab-based recordings. Thus, our findings do not indicate that community EEG recordings are of lower quality; rather, they suggest that community EEG is at least comparable, and even slightly outperformed lab-based EEG on some practical metrics.

Beyond practical data considerations, we evaluated spectral power characteristics to assess whether neural signal properties were comparable across settings. Group comparisons revealed no significant differences in power estimates across frequency bands or scalp regions, with Bayesian analyses largely supporting the null hypothesis. To complement group-level analyses, we assessed individual-level consistency in spectral power measurements using ICCs for both consistency and absolute agreement. Most region-by-band combinations demonstrated good to excellent reliability across conditions; however, this was not true for all metrics. Several low-frequency measures, such as parietal delta power, showed substantially reduced reliability. These findings suggest that while neural signal properties are broadly preserved across settings, certain metrics (particularly in lower frequency ranges) may be more sensitive to contextual or developmental variability.

Although some variability was observed across specific measures, the overall pattern of our results strongly supports the comparability of EEG recordings collected in community and laboratory settings. Collectively, these findings demonstrate that portable, community-based EEG offers a viable, flexible, and scalable alternative to traditional lab-based methods. By demonstrating similar data characteristics across lab and community EEG recordings, our study builds on previous research that has established the feasibility of leveraging portable EEG systems in developmental populations^30,31^ and comparable signal characteristics in adults^24,27^. However, our study fills a critical gap by directly comparing lab and community EEG data in young, neurodevelopmentally diverse children. This is particularly relevant for early childhood research, as this period represents a critical window for detecting and intervening in atypical developmental trajectories.

Moreover, the diversity of our cohort highlights the value of community-based EEG in reaching underrepresented populations. Traditional lab-based research can present logistical barriers, especially for those who face challenges related to transportation, time constraints, and financial costs^17,18^. These barriers are amplified in lower-income communities, where time constraints and financial concerns are often cited as obstacles to participation^18,19^. By reducing logistical barriers, community-based EEG has the potential to offer broader participation opportunities, enhancing sample diversity, generalizability, and understanding of early brain development across different social and environmental contexts.

### Limitations & next steps

Although this study provides encouraging evidence for the feasibility of community-based EEG collection, several limitations should be noted. Despite consistent findings across metrics, the modest sample size limits the ability to detect subtle effects and may not fully capture the variability in EEG responses across broader populations. As such, these results should be viewed as foundational, offering initial support for the use of portable EEG and informing the design of larger-scale studies. Additionally, while our findings demonstrate the feasibility and signal quality of community-based EEG during task-free, resting-state recordings, they may not generalize to task-based paradigms, which require greater experimental control and timing precision. Future research should evaluate the performance of community-based EEG in specific task-based contexts and identify best practices for broader application.

Finally, while community-based EEG offers a promising approach to increasing research accessibility, it is not a standalone solution to broader underrepresentation issues. Barriers to participation extend beyond logistical challenges and include systemic factors such as historical mistrust of research institutions, privacy concerns, and cultural differences in research engagement. These factors contribute to the persistent underrepresentation of certain racial, ethnic, and socioeconomically marginalized groups in research^16,17,47,48^. This is reflected in our sample, where one family declined a lab visit due to concerns about participating in research at a formal institution.

As we expand the use of community-based research, it is crucial to recognize that research participation preferences are not uniform^15,18^. While some families may find community-based EEG more accessible, others may have concerns about privacy, confidentiality, or researcher presence in the home, particularly in communities where trust in research has been historically eroded^15^. The importance of flexibility is evident in our sample, where two families chose to participate in community settings rather than at home, a request we were able to accommodate. This highlights the need for adaptive research models that promote inclusivity by responding to the individual needs and preferences of families.

## Conclusions

In conclusion, this study underscores the promise of community-based EEG as a viable and effective method for collecting high-quality neural data in developmental populations. By demonstrating signal quality comparable to that of lab-based recordings, this approach has the potential to broaden the scope of neuroscience research beyond traditional laboratory environments, thereby enhancing accessibility and inclusivity. Furthermore, it paves the way for future investigations examining children in more naturalistic settings, addressing questions related to real-world interactions and environmental engagement that are challenging to replicate in traditional lab environments. Moving forward, integrating community-driven strategies alongside community-based EEG may help to enhance the representativeness of our studies and ensure that our findings have greater relevance and impact for understudied and underserved populations.

## Data Availability

The data supporting this study will be made publicly available on the National Database of Autism Research (NDA) in accordance with the study’s timeline and data sharing policies. For inquiries regarding data access, please contact the corresponding author.

## References

[1] Khazipov, R. & Luhmann, H. J. Early patterns of electrical activity in the developing cerebral cortex of humans and rodents. *Trends Neurosci.* **29**, 414–418 (2006). 16713634 10.1016/j.tins.2006.05.007

[2] Uhlhaas, P. J. et al. Neural synchrony and the development of cortical networks. *Trends Cogn. Sci.* **14**, 72–80 (2010). 20080054 10.1016/j.tics.2009.12.002

[3] Marshall, P. J., Bar-Haim, Y. & Fox, N. A. Development of the EEG from 5 months to 4 years of age. *Clin. Neurophysiol.* **113**, 1199–1208 (2002). 12139998 10.1016/s1388-2457(02)00163-3

[4] Saby, J. N. & Marshall, P. J. The utility of EEG band power analysis in the study of infancy and early childhood. *Dev. Neuropsychol.* **37**, 253–273 (2012). 22545661 10.1080/87565641.2011.614663 PMC3347767

[5] Fogtmann, E. P. et al. Prognostic accuracy of electroencephalograms in preterm infants: A systematic review. *Pediatrics* **139**, e20161951 (2017). 28143915 10.1542/peds.2016-1951

[6] Sandoval Karamian, A. G. & Wusthoff, C. J. Current and future uses of continuous EEG in the NICU. *Front. Pediatr.* **9**, 768670 (2021). 34805053 10.3389/fped.2021.768670 PMC8595393

[7] Poil, S.-S. et al. Age dependent electroencephalographic changes in attention-deficit/hyperactivity disorder (ADHD). *Clin. Neurophysiol.* **125**, 1626–1638 (2014). 24582383 10.1016/j.clinph.2013.12.118

[8] Tierney, A. L. et al. Developmental trajectories of resting EEG power: An endophenotype of autism spectrum disorder. *PLoS ONE* **7**, e39127 (2012). 22745707 10.1371/journal.pone.0039127 PMC3380047

[9] Wang, J. et al. Resting state EEG abnormalities in autism spectrum disorders. *J. Neurodev. Disord.* **5**, 24 (2013). 24040879 10.1186/1866-1955-5-24 PMC3847481

[10] Dickinson, A. et al. Early patterns of functional brain development associated with autism spectrum disorder in tuberous sclerosis complex. *Autism Res*10.1002/aur.2193 (2019). 31419043 10.1002/aur.2193 PMC6898751

[11] Frohlich, J. et al. Mechanisms underlying the EEG biomarker in Dup15q syndrome. *Mol. Autism* **10**, 29 (2019). 31312421 10.1186/s13229-019-0280-6 PMC6609401

[12] Sidorov, M. S. et al. Delta rhythmicity is a reliable EEG biomarker in Angelman syndrome: a parallel mouse and human analysis. *J. Neurodev. Disord.* **9**, 17 (2017). 28503211 10.1186/s11689-017-9195-8 PMC5422949

[13] Shuffrey, L. C. et al. Association between prenatal exposure to alcohol and tobacco and neonatal brain activity: Results from the safe passage study. *JAMA Netw. Open* **3**, e204714 (2020). 32396193 10.1001/jamanetworkopen.2020.4714 PMC7218492

[14] McLaughlin, K. A. et al. Adverse rearing environments and neural development in children: The development of frontal electroencephalogram asymmetry. *Biol. Psychiatry* **70**, 1008–1015 (2011). 21962332 10.1016/j.biopsych.2011.08.006 PMC3207006

[15] Alvarado, F. et al. Qualitative analysis of stakeholder perspectives on engaging Latinx patients in kidney-related research. *BMC Nephrol.* **24**, 79 (2023). 36991364 10.1186/s12882-023-03128-y PMC10061843

[16] Bardach, S. H. et al. Insights from African American older adults on brain health research engagement: “need to see the need”. *J. Appl. Gerontol.* **40**, 201–208 (2021). 32013658 10.1177/0733464820902002 PMC7398819

[17] Luebbert, R. & Perez, A. Barriers to clinical research participation among African Americans. *J. Transcult. Nurs.* **27**, 456–463 (2016). 25754929 10.1177/1043659615575578

[18] Occa, A., Morgan, S. E. & Potter, J. E. Underrepresentation of Hispanics and other minorities in clinical trials: recruiters’ perspectives. *J. Racial Ethn. Health Dispar.* **5**, 322–332 (2018). 10.1007/s40615-017-0373-x 28452008

[19] Tanner, A. et al. Barriers to medical research participation as perceived by clinical trial investigators: communicating with rural and African American communities. *J. Health Commun.* **20**, 88–96 (2015). 25204763 10.1080/10810730.2014.908985

[20] Deoni, S. C. L. et al. Remote and at-home data collection: Considerations for the NIH HEALthy brain and cognitive development (HBCD) study. *Dev. Cogn. Neurosci.* **54**, 101059 (2022). 35033972 10.1016/j.dcn.2022.101059 PMC8762360

[21] Sugden, R. J. et al. Remote collection of electrophysiological data with brain wearables: opportunities and challenges. *Bioelectron. Med.* **9**, 12 (2023). 37340487 10.1186/s42234-023-00114-5 PMC10283168

[22] Hervé, E. et al. Challenges and new perspectives of developmental cognitive EEG studies. *Neuroimage* **260**, 119508 (2022). 35882267 10.1016/j.neuroimage.2022.119508

[23] Van Diessen, E. et al. Opportunities and methodological challenges in EEG and MEG resting state functional brain network research. *Clin. Neurophysiol.* **126**, 1468–1481 (2015). 25511636 10.1016/j.clinph.2014.11.018

[24] Edwards, D. J. & Trujillo, L. T. An analysis of the external validity of EEG spectral power in an uncontrolled outdoor environment during default and complex neurocognitive states. *Brain Sci.* **11**, 330 (2021). 33808022 10.3390/brainsci11030330 PMC7998369

[25] Marini, F. et al. A comparative evaluation of signal quality between a research-grade and a wireless dry-electrode mobile EEG system. *J. Neural Eng.* **16**, 054001 (2019). 31096191 10.1088/1741-2552/ab21f2

[26] Melnik, A. et al. Systems, subjects, sessions: To what extent do these factors influence EEG data?. *Front. Hum. Neurosci.* **11**, 150 (2017). 28424600 10.3389/fnhum.2017.00150 PMC5371608

[27] Mikkelsen, K. B. et al. EEGs vary less between lab and home locations than they do between people. *Front. Comput. Neurosci.* **15**, 565244 (2021). 33679356 10.3389/fncom.2021.565244 PMC7928278

[28] Bhavnani, S. et al. The acceptability, feasibility, and utility of portable electroencephalography to study resting-state neurophysiology in rural communities. *Front. Hum. Neurosci.* **16**, 802764 (2022). 35386581 10.3389/fnhum.2022.802764 PMC8978891

[29] Estrin GL, Bhavnani S, Goodwin A, et al. From the lab to the field: acceptability of using electroencephalography with Indian preschool children, *Wellcome Open Res*, **7**, 99. 10.12688/wellcomeopenres.17334.2 (2023). 10.12688/wellcomeopenres.17334.2 PMC10632594 37953927

[30] Giannadou, A. et al. Investigating neural dynamics in autism spectrum conditions outside of the laboratory using mobile electroencephalography. *Psychophysiology*10.1111/psyp.13995 (2022). 34982474 10.1111/psyp.13995

[31] Lau-Zhu, A., Lau, M. P. H. & McLoughlin, G. Mobile EEG in research on neurodevelopmental disorders: Opportunities and challenges. *Dev. Cogn. Neurosci.* **36**, 100635 (2019). 30877927 10.1016/j.dcn.2019.100635 PMC6534774

[32] Troller-Renfree, S. V. et al. Feasibility of assessing brain activity using mobile, in-home collection of electroencephalography: methods and analysis. *Dev. Psychobiol.*10.1002/dev.22128 (2021). 34087950 10.1002/dev.22128 PMC8478406

[33] Delorme, A. & Makeig, S. EEGLAB: an open source toolbox for analysis of single-trial EEG dynamics including independent component analysis. *J. Neurosci. Methods* **134**, 9–21 (2004). 15102499 10.1016/j.jneumeth.2003.10.009

[34] Levin, A. R. et al. EEG power at 3 months in infants at high familial risk for autism. *J. Neurodev. Disord.* **9**, 1–13 (2017). 28903722 10.1186/s11689-017-9214-9 PMC5598007

[35] Dickinson, A. et al. Interhemispheric alpha-band hypoconnectivity in children with autism spectrum disorder. *Behav. Brain Res.* **348**, 227–234 (2018). 29689375 10.1016/j.bbr.2018.04.026 PMC5993636

[36] Chang C, Hsu S, Pion-Tonachini L, et al. Evaluation of Artifact Subspace Reconstruction for Automatic EEG Artifact Removal. In: *2018 40th Annual International Conference of the IEEE Engineering in Medicine and Biology Society (EMBC)*, 1242–1245. (2018). 10.1109/EMBC.2018.8512547 30440615

[37] Cohenour, T. et al. Patterns of spontaneous neural activity associated with social communication abilities among infants and toddlers showing signs of autism. *Eur. J. Neurosci.* **60**, 3597–3613 (2024). 38703054 10.1111/ejn.16358 PMC12083214

[38] Dickinson, A. et al. Multivariate neural connectivity patterns in early infancy predict later autism symptoms. *Biol. Psychiatry Cogn. Neurosci. Neuroimaging.* **6**, 59–69 (2021). 32798139 10.1016/j.bpsc.2020.06.003 PMC7736067

[39] Dickinson, A. et al. Multi-site EEG studies in early infancy: Methods to enhance data quality. *Dev. Cogn. Neurosci.* **69**, 101425 (2024). 39163782 10.1016/j.dcn.2024.101425 PMC11380169

[40] Lopez-Calderon, J. & Luck, S. J. ERPLAB: an open-source toolbox for the analysis of event-related potentials. *Front. Hum. Neurosci.* **8**, 213 (2014). 24782741 10.3389/fnhum.2014.00213 PMC3995046

[41] Xie, W., Toll, R. T. & Nelson, C. A. EEG functional connectivity analysis in the source space. *Dev. Cogn. Neurosci.* **56**, 101119 (2022). 35716637 10.1016/j.dcn.2022.101119 PMC9204388

[42] Gudmundsson, S. et al. Reliability of quantitative EEG features. *Clin. Neurophysiol.* **118**, 2162–2171 (2007). 17765604 10.1016/j.clinph.2007.06.018

[43] Dickinson, A. et al. Peak alpha frequency is a neural marker of cognitive function across the autism spectrum. *Eur. J. Neurosci.*10.1111/ejn.13645 (2017). 28700096 10.1111/ejn.13645 PMC5766439

[44] Rouder, J. N. et al. Bayesian t tests for accepting and rejecting the null hypothesis. *Psychon. Bull. Rev.* **16**, 225–237 (2009). 19293088 10.3758/PBR.16.2.225

[45] Liljequist, D., Elfving, B. & Skavberg, R. K. Intraclass correlation–A discussion and demonstration of basic features. *PLoS ONE* **14**, e0219854 (2019). 31329615 10.1371/journal.pone.0219854 PMC6645485

[46] Koo, T. K. & Li, M. Y. A guideline of selecting and reporting intraclass correlation coefficients for reliability research. *J. Chiropr. Med.* **15**, 155–163 (2016). 27330520 10.1016/j.jcm.2016.02.012 PMC4913118

[47] Darnell, K. R., McGuire, C. & Danner, D. D. African American participation in Alzheimer’s disease research that includes brain donation. *Am. J. Alzheimers Dis. Dement.* **26**, 469–476 (2011). 10.1177/1533317511423020 PMC3329867 22009227

[48] George, S., Duran, N. & Norris, K. A systematic review of barriers and facilitators to minority research participation among African Americans, Latinos, Asian Americans, and Pacific Islanders. *Am. J. Public Health* **104**, e16–e31 (2014). 24328648 10.2105/AJPH.2013.301706 PMC3935672

